# Detrimental effects of prior self‐control exertion on subsequent sporting skill performance

**DOI:** 10.1111/sms.14011

**Published:** 2021-07-06

**Authors:** Ruth Boat, Caroline Sunderland, Simon B. Cooper

**Affiliations:** ^1^ Sport Science Department Sport Health and Performance Enhancement (SHAPE) Research Centre School of Science and Technology Nottingham Trent University Nottingham UK

**Keywords:** cognition, cognitive exertion, cortisol, ego depletion, field hockey, sports performance, sustained attention

## Abstract

The prior exertion of self‐control has previously been shown to negatively affect physical performance, yet the effects on complex sporting skill performance have not been examined. Therefore, this study examined whether prior self‐control exertion influences performance on a field hockey task, alongside measuring plasma cortisol concentration and attention as potential mechanisms to explain any effects.

Following familiarization, 13 male hockey players (20 ± 1 years) participated in a randomized, order‐balanced, crossover design. For the manipulation of self‐control, participants completed an incongruent (self‐control exertion trial) or a congruent (control trial) Stroop task. Skill performance was assessed using a field hockey skills task. Capillary blood samples, for the determination of plasma cortisol concentration, were taken at baseline, post‐Stroop task, and post‐field hockey skills task. Cognitive tests of attention (RVIP and Flanker tasks) were completed following the field hockey skills task.

Participants made more errors in the latter stages of the field hockey skills task following self‐control exertion (trial*time interaction, *p* = 0.041). Participants also made more errors on the RVIP task following self‐control exertion (*p* = 0.035); yet the time taken to complete the hockey skills task, performance on the flanker task, and plasma cortisol concentrations were unaffected (all *p* > 0.05).

Overall, these findings suggest that prior self‐control exertion has detrimental effects on subsequent sporting skill performance (more errors made on the field hockey task), which may be explained by poorer sustained attention (lower accuracy on the RVIP task). This suggests that athletes should aim to avoid self‐control exertion before a competitive match to optimize performance.

## 1 INTRODUCTION

Self‐control refers to abilities that enable individuals to exert control over their behaviors, emotions, thoughts, and to pursue their goals^1^; and is a fundamental feature of executive function, including the sub‐domains of inhibitory control and cognitive flexibility.^2^, ^3^, ^4^ High levels of trait self‐control are believed to be important for physical activity behavior^5^ and are associated with a stronger relationship between physical activity intentions and behaviors.^4^ Self‐control has been viewed as a specific form of self‐regulation, in which an individual exerts deliberate and conscious effort to control the self, whereas self‐regulation is considered a global term that encapsulates automatic and nonconscious regulatory processes.^1^ Specifically, self‐control is linked to many positive behavioral outcomes,^6^ including sport and exercise performance.^7^ For instance, individuals are required to successfully perform complex, sport‐specific, skill‐based tasks (eg, in sports such as basketball, soccer, and hockey), which necessitates the control of one's emotional, cognitive, and motor processes.^8^

Self‐control capacity can vary between individuals (ie, trait self‐control), as well as within individuals across situations (ie, state self‐control).^9^ For instance, some researchers posit that the resources responsible for self‐control are finite and become diminished when an individual regulates his or her behaviors, a state commonly termed “ego depletion.”^1^ Accordingly, the individual will have a reduced capability to perform any subsequent behavior that requires self‐control, with this “limited resource” perspective having received meta‐analytic support.^10^ However, some researchers have challenged this resource explanation. For example, studies have shown that when participants were in a state of ego depletion, performance on a subsequent task was not impaired if they were adequately motivated, using techniques such as providing incentives^11^ and offering choice.^12^ This raises doubts regarding the idea that self‐control failure is the result of a resource that becomes depleted.^13^ As a result of theoretical criticisms, other researchers have postulated the shifting priorities model; suggesting that following the exertion of self‐control individuals experience shifts in motivation and attention that undermine performance on subsequent tasks that also require self‐control.^14^, ^15^

Despite the ongoing theoretical debate regarding the processes governing self‐control, meta‐analytic evidence has demonstrated that following the exertion of state self‐control on one task, performance on a subsequent, ostensibly unrelated physical task requiring self‐control, is reduced.^16^, ^17^ For example, individuals who completed a task requiring self‐control (incongruent Stroop task) were unable to sustain an isometric handgrip squeeze for as long, compared with individuals who completed a task requiring no self‐control (congruent Stroop task).^18^, ^19^ Squeezing a handgrip essentially requires muscular endurance, yet overcoming fatigue or pain and superseding the desire to quit are acts that necessitate self‐control.^20^ However, the existence of this depletion effect has been questioned,^21^ with a Registered Replication Report not finding support for the negative effects of prior self‐control exertion on subsequent tasks also requiring self‐control.^22^ Consequently, many commentaries and debates have implied that publication bias may have led to an overestimation of the size of the depletion effect.^21^, ^23^

Building on this literature and to address the replication crisis, researchers have begun to improve the ecological validity of the findings, in an attempt to formulate conclusions regarding more complex sports performance. For instance, depletion effects have been demonstrated for effort‐based physical tasks, such as cycling performance,^24^, ^25^ wall‐sit task performance,^26^, ^27^ and press‐up and sit‐up performance.^28^ Evidently, self‐control exertion appears to impair subsequent performance that requires prolonged effort. In addition, from a mental fatigue perspective, research has found that when participants completed a 90‐min demanding cognitive task (the AX‐Continuous Performance Task) perceived exertion was significantly higher, and participants disengaged earlier, during a subsequent cycling task to exhaustion at 80% peak power output, compared with when they completed a control task (90 min of watching emotionally neutral documentaries).^29^ It is important to note, however, there are some significant differences between self‐control exertion and mental fatigue.^7^ Typically, tasks that are employed within the mental fatigue literature last substantially longer (~90 min), compared with the tasks that are utilized in self‐control depletion research (~4–10 min). Despite the differences, both self‐control depletion and mental fatigue seem to lead to performance decrements on physical tasks that require prolonged effort.^16^

Regarding self‐control depletion, researchers have begun to explore the effects of self‐control exertion on subsequent skill‐based sports tasks, which require numerous self‐regulation behaviors, including regulating one's attention, emotions, and motor skill execution.^30^ For instance, following a task requiring self‐control (incongruent Stroop task), participants demonstrated poorer accuracy on a dart‐throwing task, compared with when they completed a task requiring no self‐control (congruent Stroop task).^30^ The ability for self‐control exertion to affect subsequent skill‐based performance has been corroborated during basketball free‐throw shooting tasks,^8^, ^31^ as well as a golf‐putting task.^32^

Although these studies provide valuable insight into the effects of self‐control exertion on subsequent skill‐based performance, it is currently unknown whether self‐control exertion impairs more complex skill‐based performance; for example in team sports where skill performance is a key determinant of success.^30^ Furthermore, the underpinning physiological mechanisms which may explain the effects of self‐control exertion on subsequent sporting performance remain unknown. Glucose has been proposed as a potential resource within the limited resource model of self‐control, with initial research highlighting that exerting self‐control reduced blood glucose, leading to impaired performance on subsequent tasks.^33^ Furthermore, consuming glucose‐based drinks has attenuated the reductions in performance following the exertion of self‐control.^34^ However, recent findings have not found support for the moderating role of glucose,^24^, ^35^, ^36^ raising doubts as to whether glucose is the resource that governs self‐control.^37^, ^38^

Subsequently, it is speculated that cortisol may have a role to play, given that cortisol has recently been shown to be elevated to a greater extent following combined mental and physical exertion, when compared to physical exertion alone^39^ and following a task (30‐min Stroop task) designed to induce mental fatigue.^40^ Furthermore, among numerous physiological effects,^41^ elevated cortisol levels have been shown to negatively affect subsequent cognitive performance, in particular attention.^42^ This is an important consideration as attention is a key cognitive domain for successful sporting skill performance,^43^ and is one of the proposed mechanisms within the shifting priorities model of self‐control.^14^, ^15^ Indeed, previous research has demonstrated decrements in visual attention (gaze behavior) following the exertion of self‐control,^44^ yet this has not been examined in a sport‐specific context. Thus, to our knowledge, no studies to date have examined the effects of self‐control exertion on cortisol or cognitive tests of attention in a sporting context; both of which could explain any subsequent effects upon sporting skill performance.

Therefore, the primary aim of the present study was to determine whether self‐control exertion influences subsequent sporting skill performance on a field hockey task. Furthermore, a secondary aim of the study was to examine whether self‐control exertion affects plasma cortisol concentration and subsequent attention, to consider whether these may be mechanisms that explain any effects on subsequent sporting skill performance. Based on the effect of self‐control exertion on isolated skill performance,^30^ it was hypothesized that self‐control exertion would result in reduced performance on the field hockey skills test (hypothesis 1). Furthermore, it is hypothesized that plasma cortisol levels would increase (hypothesis 2), and performance on tests of attention would be lower (hypothesis 3), following the exertion of self‐control.

## 2 MATERIALS AND METHODS

### 2.1 Participants and study design

Following approval from the institution's ethical advisory committee, 13 sub‐elite (competing in national or regional competitive leagues) male hockey players (age: 20 ± 1 years old; height: 179.3 ± 5.5 cm; body mass: 78.4 ± 4.3 kg) volunteered to take part in this study. All participants provided their written informed consent to participate and had no medical condition that could affect their participation in the study, as determined by the University‐approved health screen questionnaire, which assessed physical, psychological, and neurological health. The study employed a randomized, order‐balanced, single‐blind, crossover design. Following two familiarization sessions, participants completed two main experimental trials (self‐control exertion and control), each separated by at least 48 h.

A power calculation (G*Power, version 3.1),^45^ based on repeated measures ANOVA (within factors, power = 0.95, α = 0.05) specified that a minimum sample size of *n* = 12 would be satisfactory to detect a medium effect size (0.50), which is representative of other skill‐based self‐control studies.^30^

### 2.2 Experimental protocol

During the first familiarization session, the experimental protocol and measures were explained to the participants in full and they were provided an opportunity to ask any questions. Following this, participants were provided with an opportunity to practice the hockey skills test and cognitive function tests; a further opportunity to practice these tests was provided at the second familiarization session. Two familiarization sessions have previously been recommended when using the hockey skills test, to enhance reliability and eliminate any potential learning effect.^46^

Prior to each main experimental trial, participants were asked to refrain from strenuous physical activity and alcohol consumption for 24 h. All testing took place in a University sports hall. Upon arrival to the testing session, participants completed a daily stress and physical fatigue questionnaire. Daily stress was measured using seven items from the Daily Inventory of Stressful Events questionnaire,^47^ and physical fatigue was measured using two items from the fatigue subscale of the Profile of Mood States (POMS) questionnaire.^48^ Both of these measures have been used successfully to control for daily stress and fatigue in self‐control studies of a similar nature.^26^

Following these baseline measurements, participants completed a 5‐min self‐selected warm‐up, followed by a capillary blood sample (see section 2.6) for the determination of plasma cortisol concentration. Following this, participants completed the Stroop task, which was used in the present study to manipulate self‐control (see section 2.3). Immediately following the Stroop task, participants completed the CR‐10 scale to rate their mental exertion (see section 2.3.1) and had a further capillary blood sample taken, before commencing the hockey skills task (see section 2.4). Immediately following the hockey skills task, participants had a final capillary blood sample taken and then completed two tests of attention (Flanker task and Rapid Visual Information Processing (RVIP) task; see section 2.5).

### 2.3 Manipulation of self‐control

Self‐control was manipulated using a Stroop task, in line with many previous self‐control exertion studies.^27^, ^49^ In brief, participants completed the congruent Stroop task on the control trial and the incongruent Stroop task on the self‐control exertion trial. Both Stroop tasks involved a central “stimulus” word being presented on the screen, with the target (correct answer) on one side of the screen and a distractor (incorrect answer) on the other side. For the congruent Stroop task, the target word and the font color were congruent (eg, “red” written in red font). On the incongruent Stroop task, the target word and font color were incongruent (eg, “red” written in green font), and the participant had to override their dominant response of selecting the word itself, and select the font color instead (in this example, the correct response would be green). The Stroop task was presented on a laptop computer using custom‐made software, which participants viewed at a head‐to‐monitor distance of 80–100 cm; with participants using the keyboard arrow keys to make their responses. Both Stroop tasks contained six practice stimuli where feedback was provided on whether the response was correct or not; followed by 160 stimuli (lasting approximately 4 min), with each stimulus remaining on the screen until a response was registered. This duration of the Stroop tasks was employed as previous research has successfully employed this task for the same length of time (ie, 4 min).^26^, ^27^, ^50^

#### 2.3.1 Manipulation checks

The singe item Borg CR‐10 scale^51^ was completed following the Stroop task to measure mental exertion. Participants respond by selecting a number between 0 (“extremely weak”) and 10 (“absolute maximum”) to indicate their mental exertion on the Stroop task. This is a commonly used manipulation check in self‐control research.^26^, ^27^, ^50^

### 2.4 Hockey skills test

The hockey skills test utilized in the present study was the field hockey skill test of Sunderland et al. (2006),^46^ which has previously been shown as having excellent testretest reliability (*r* = 0.96) and validity (*r* = 0.61–0.83), comparing favorably to alternative tests of sporting skill performance.^52^ In brief, the hockey skill test required participants to start from a line 16 yards from a standard hockey goal, before dribbling round a series of cones, making a pass against a rebound board and then shooting to either the left or right side of the goal. At the completion of the dribbling phase, the players break an infra‐red beam which triggers a light on either side of the goal (left or right) and start the “decision‐making time” timer. The participant must shoot to the opposite side of the goal to the light that is illuminated, with the decision‐making time stopped by an automatic sound‐based trigger which detects the ball hitting the goal. The player then runs back to the start line; with each set consisting of six repeats of the task. Participants completed four sets (each of six repeats), with each set separated by 90 s rest. If any errors were made (eg, touching a cone when dribbling, missing the rebound board, or missing the target), an error was recorded (which also adds 2 s, per error, to the overall performance time). The variables of interest were total performance time (including error penalties), average decision‐making time, and the number of errors made. For a full description of the hockey skill test design and implementation, please refer to Sunderland et al. (2006).^46^ This hockey skills test requires numerous self‐control behaviors, including the regulation of attention, emotions, and motor skill execution. For instance, during the test, participants are required to use their self‐control to inhibit their immediate desire to shoot to the side of the goal where the light is illuminated, but instead to shoot to the opposite side of the goal to the light that is illuminated. In addition, the hockey skills test requires the execution of complex motor skills in a high‐pressure situation,^53^ which also requires self‐control.

### 2.5 Tests of attention

Following completion of the hockey skill task, participants completed two tests of attention; the Flanker task and RVIP task. These tests were administered using custom‐made software on a laptop computer and are described in detail elsewhere (Flanker task^54^; RVIP task^55^). In brief, the Flanker task is a test of attention^56^ and required participants to select the direction of a central arrow on congruent (ie, all arrows pointing in the same direction, eg, < < < < <) and incongruent (ie, central arrow points in a different direction, eg, < < > < <) stimuli. Participants responded using the arrow keys on the laptop computer and the test consisted of 60 stimuli in total (30 congruent and 30 incongruent, in a random order). The RVIP task^55^ is a test of sustained attention (5‐min duration) and required participants to monitor a stream of digits (2–9), presented at a rate of 100 digits·min^−1^ on a laptop screen, and identify target sequences of 3 odd or even numbers (eg, 2–8–6, 7–3–9 etc.) by pressing the space bar. A correct response could be registered during the presentation of the last digit of a target and the following 1500 ms. For both the Flanker and RVIP tasks, the variables of interest were the response times of correct responses and the proportion of correct responses made.

### 2.6 Capillary blood samples

For the determination of plasma cortisol concentration, capillary blood samples were taken. A single‐use lancet (Unistik, Extra, 21G gauge, 2.0 mm depth, Owen Mumford Ltd, UK) was used and the blood was collected into a 300 μl EDTA‐coated microvette (Sarstedt Ltd, UK). The sample was then centrifuged at 1000 g for 4 min at 4°C (Eppendorf 5415C, Hamburg, Germany) and the plasma pipetted into a 500 μl plastic vial, before being frozen at −80°C until subsequent analysis. Plasma cortisol concentration was determined in singular using a commercially available kit (ELISA, R&D Systems Europe Ltd., UK), with an intra‐assay coefficient of variation of 11.3% based on eight repeat measurements.

### 2.7 Statistical analysis

Data were analyzed using SPSS (version 26; SPSS Inc., Chicago, IL, USA). To examine baseline differences between the trials and confirm the manipulation of self‐control, daily stress, physical fatigue, and mental exertion were compared between the trials using a paired‐samples *t* test, with effect sizes reported as Cohen's *d*, interpreted as per convention (ie, small: 0.2; medium: 0.5; large: 0.8).

The outcome variables (total performance time, decision‐making time, and number of errors) from the hockey skills test were examined using two‐way (trial [self‐control exertion vs. control] * set [set 1, 2, 3 and 4]) repeated measures analysis of variance (ANOVA), with effect sizes reported as partial eta squared (*η_p_* ^2^) interpreted as per convention (ie, small: 0.01; medium: 0.06; large: 0.14). Where a significant trial * set interaction existed, post‐hoc analyses were conducted using Bonferroni corrected paired‐samples *t* tests. Plasma cortisol concentration was also assessed using a two‐way (trial * time) repeated measures ANOVA. Response times and accuracy from the initial Stroop test and subsequent tests of attention were compared between the trials using paired‐samples t tests, with effect sizes reported as Cohen's *d*. All data are reported as mean ± standard deviation, and statistical significance was accepted as *p* ≤ 0.05.

## 3 RESULTS

### 3.1 Pre‐trial manipulation checks

There was no difference in baseline stress (*p* = 0.700) or fatigue (*p* = 0.104) between the self‐control exertion and control trials. However, the manipulation of self‐control was successful in affecting mental exertion, as assessed via the CR‐10. Specifically, participants reported greater mental exertion following the incongruent Stroop task on the self‐control exertion trial, compared with following the congruent Stroop task on the control trial (self‐control exertion: 4.2 ± 1.4, control: 2.8 ± 1.4; *t*_(12)_ = 3.5, *p* = 0.005, *d* = 1.0). This was confirmed with differences in Stroop test performance between the self‐control exertion and control trials, whereby participants responded slower (self‐control exertion: 1958 ± 310 ms, control: 1584 ± 261 ms; *t*_(12)_ = 3.8, *p* < 0.001, *d* = 1.31) and with lower accuracy (self‐control exertion: 96.2 ± 3.8%, control: 97.8 ± 2.1%; *t*_(12)_ = −2.9, *p* = 0.016, *d* = 0.54), following self‐control exertion.

### 3.2 Hockey skill test performance

The total performance time, decision‐making time, and number of errors across the four repeats of the hockey skill test on both the self‐control exertion and control trials can be seen in Table 1.

**TABLE 1:** Hockey skill performance test across the self‐control exertion and control trials. Data are mean ± SD

Variable	Trial	Set				Overall
		1	2	3	4	
Total performance time [s]	Self‐control exertion	105.4 ± 12.3	100.8 ± 8.7	100.6 ± 10.3	101.1 ± 8.3	407.9 ± 39.6
	Control	109.7 ± 12.0	102.2 ± 9.4	99.6 ±10.9	101.3 ± 12.1	412.8 ± 44.4
Decision‐making time [s]	Self‐control exertion	5.78 ± 0.94	5.70 ± 1.07	5.65 ± 1.20	5.84 ± 1.40	23.0 ± 4.60
	Control	5.83 ± 1.18	5.39 ± 0.73	5.28 ± 0.71	5.29 ± 0.82	21.8 ± 3.44
Errors [*n*]	Self‐control exertion	4.8 ± 1.4	5.5 ± 1.5	5.9 ± 2.1*	6.2 ± 2.2*	22.4 ± 5.0*
	Control	5.0 ± 1.6	4.7 ± 1.4	3.9 ± 1.3	4.4 ± 1.7	18.0 ± 4.5

* Significant difference between trials (*p* < 0.05).

#### 3.2.1 Total performance time

Overall, there was no difference in total performance time between the self‐control exertion and control trials (main effect of trial, *p* = 0.566), although total performance time did improve on both trials across the four sets (main effect of time, *F*_(3,36)_ = 9.5, *p* < 0.001, *η_p_* ^2^ = 0.443). However, the pattern of change in total performance time across the four sets was not different between the self‐control exertion and control trials (trial * time interaction, *p* = 0.553).

#### 3.2.2 Decision‐making time

Overall, there was also no difference in decision‐making time between the self‐control exertion and control trials (main effect of trial, *p* = 0.301), nor did decision‐making time change across the four sets (main effect of time, *p* = 0.441). Furthermore, the pattern of change in decision‐making time across the four sets was the same between the self‐control exertion and control trials (trial * time interaction, *p* = 0.490).

#### 3.2.3 Errors

**FIGURE 1:**
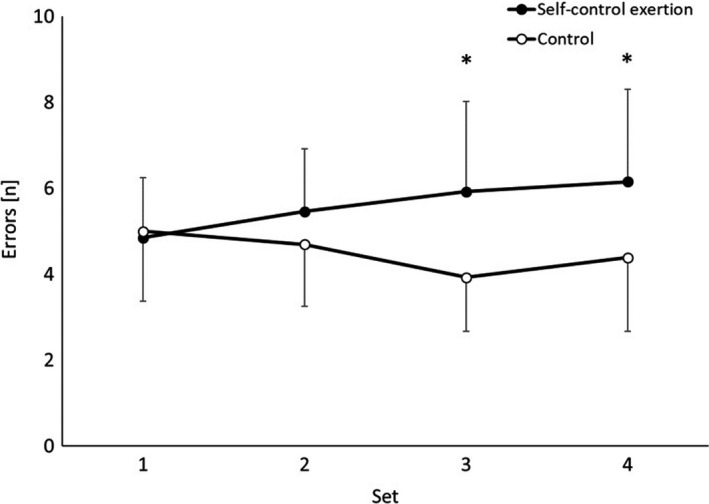
Number of errors made (mean ±SD) across the 4 sets of the hockey skill test on the self‐control exertion and control trials (main effect of trial, *p* = 0.014; trial * time interaction, *p* = 0.041; *self‐control exertion >control, *p* < 0.05).

Overall, participants made more errors, per set, on the self‐control exertion trial, compared with the control trial (self‐control exertion: 5.6 ± 1.2, control: 4.5 ± 1.1; main effect of trial, *F*_(1,12)_ = 8.2, *p* = 0.014, *η_p_* ^2^ = 0.405); yet the number of errors did not change across the four sets of the test (main effect of time, *p* = 0.733). However, there was a significant trial * time interaction, whereby participants made more errors in the latter sets of the hockey skill test following self‐control exertion, when compared to the control trial (trial * time interaction, *F*_(3,36)_ =3.1, *p* = 0.041, *η_p_* ^2^ = 0.202; Figure 1). Specifically, following self‐control exertion, participants made more errors on set 3 (*t*_(12)_ = 3.0, *p* = 0.011, *d* = 1.18) and set 4 (*t*_(12)_ = 2.8, *p* = 0.017, *d* = 0.92), when compared to the control trial.

### 3.3 Tests of attention

Response times and accuracy for the Flanker task and RVIP task can be seen in Table 2.

**TABLE 2:** Response times and accuracy for the Flanker and RVIP tasks on the self‐control exertion and control trials. Data are mean ± SD.

Test	Variable	Test level	Self‐control exertion	Control
Flanker task	Response time [ms]	Congruent	477 ± 55	467 ± 57
		Incongruent	507 ± 65	509 ± 65
	Accuracy [%]	Congruent	99.7 ± 0.9	99.5 ± 1.8
		Incongruent	97.2 ± 4.0	96.7 ± 3.6
RVIP task	Response time [ms]	‐	510 ± 96	496 ± 96
	Accuracy [%]	‐	47.5 ± 18.5	55.0 ± 20.0 *

* ignificant difference between trials (*p* < 0.05).

#### 3.3.1 Flanker task

Response times on the Flanker task were not different between the self‐control exertion and control trials for either the congruent (*p* = 0.618) or incongruent (*p* = 0.914) stimuli. Furthermore, accuracy was also similar between the trials for both the congruent (*p* = 0.672) and incongruent (*p* = 0.657) stimuli.

#### 3.3.2 RVIP task

Response times on the RVIP task were not different between the self‐control exertion and control trials (*p* = 0.579). However, participants achieved a lower proportion of correct responses on the self‐control exertion trial when compared to the control trial (self‐control exertion: 47.5 ± 18.5%, control: 55.0 ± 20%; *t*_(12)_ = −2.4, *p* = 0.035, *d* = 0.39).

### 3.4 Plasma cortisol concentration

Overall, plasma cortisol concentration was not different between the self‐control exertion and control trials (main effect of trial, *p* = 0.216), nor did it differ across time (main effect of time, *p* = 0.213). Furthermore, the pattern of change in plasma cortisol concentration over time was similar between the self‐control exertion and control trials (trial * time interaction, *p* = 0.279).

## 4 DISCUSSION

The findings of the present study provide novel evidence that following self‐control exertion participants made more errors on the field hockey task; specifically, these errors were made toward the end of the task (in sets 3 and 4). However, there was no effect of self‐control exertion on overall performance time and decision‐making on the field hockey task. Overall, this suggests that complex sporting skill performance was detrimentally affected by prior self‐control exertion. Furthermore, performance on the RVIP task (a test of sustained attention) was lower (ie, more errors were made) following self‐control exertion; however, there was no effect on plasma cortisol concentration.

A key finding of the present study was that following a task requiring self‐control, participants made more errors on a field hockey task, relative to when they completed a task requiring no self‐control. This is in accordance with previous research using isolated skill‐based tasks,^8^, ^31^, ^32^ with the present study extending these findings to show that self‐control exertion led to impaired accuracy on a subsequent task requiring complex sporting skill‐based performance. More specifically, following a task requiring self‐control, participants made more errors toward the end of the field hockey task (ie, in sets 3 and 4), compared with when they completed a task requiring no self‐control. This finding suggests that self‐control exertion could have a greater impact on sporting skill performance in the latter stages of a competitive game. However, despite affecting accuracy on the field hockey task, prior self‐control exertion did not affect overall performance time. This finding is in line with previous research suggesting that self‐control exertion affects accuracy, but not speed, on a subsequent skill‐based task.^30^ It could also be argued that this finding is in accordance with theories of self‐control,^1^, ^14^ whereby prior self‐control exertion led to decreased self‐control in the subsequent field hockey task, which manifests as being unable or unwilling to self‐regulate accuracy toward the end of the performance task, rather than a slower performance time overall.

In addition to the effects on sporting skill performance, the findings of the present study suggest that the accuracy of sustained attention (as assessed by the RVIP task) was lower following self‐control exertion, when compared to the control trial. Conversely, performance on the Flanker task was unaffected by self‐control exertion. To the author's knowledge, this is the first study to demonstrate the detriment in sustained attention, in a sport‐specific context, following self‐control exertion, which is of interest given that attention is one of the proposed mechanisms within the shifting priorities model of self‐control.^14^, ^15^ Furthermore, attention is an important requirement for successful sporting performance;^30^ thus, the poorer accuracy of sustained attention could explain the fact that more errors were made toward the latter stages (sets 3 and 4) of the field hockey skills task. These findings are in line with evidence from mainstream psychology that prior self‐control exertion leads to decrements in subsequent visual attention.^44^ The present study extends these findings, by demonstrating that sustained attention is negatively affected following self‐control exertion; which is hypothesized to, at least in part, explain the poorer sporting skill performance. Although the findings of the current study are in line with the shifting priorities model from an attentional perspective, we did not examine the motivational aspect of this model. Recent research has revealed that motivation to perform task goals may be an important explanatory mechanism behind performance decrements on physical tasks following self‐control exertion.^27^, ^50^ Future research should make efforts to explore whether the exertion of self‐control leads to a reduction in motivation during subsequent complex sporting skill performance.

The present study is the first to examine the effects of self‐control exertion on plasma cortisol concentration. It was hypothesized that self‐control exertion would lead to an increase in plasma cortisol concentration, given that combined physical and mental exertion has been shown to result in elevated cortisol.^39^ However, there was no difference in cortisol between the self‐control exertion and control trials at any time point. Therefore, the present study provides preliminary evidence that cortisol is not an underpinning mechanism explaining the negative effects of self‐control exertion on subsequent sporting skill performance and sustained attention. However, the present study is the first to examine this, with future studies required to confirm this initial finding.

Although the findings of the present study provide novel evidence that self‐control exertion led to impaired accuracy on a subsequent task requiring complex skill‐based performance, we cannot generalize our results to other sports. Therefore, it would be valuable for other researchers to conduct similar experiments on complex skill‐based tasks in other team sports (eg, soccer). Similarly, future research could explore the effects of self‐control exertion on subsequent complex skill performance during a competitive match (eg, a hockey match); this would improve the ecological validity of our findings. However, the multitude of factors which could impact skill performance in such situations would be incredibly difficult to control. Furthermore, in the current study we utilized a 4‐min congruent and incongruent Stroop task; however, recent research has implied that engaging in longer durations of the initial self‐control task (ie, the Stroop task in the current study) leads to greater detrimental effects on subsequent physical performance.^57^ Future research could employ an initial self‐control task for a longer duration, to provide further insight into the potential for the duration of the initial self‐control task to influence subsequent complex skill‐based performance. It is also important to acknowledge that we did not measure trait self‐control in the current study. It is possible that individuals with low trait self‐control will be more susceptible to becoming depleted compared with those with high trait self‐control. This is because those with low trait self‐control may utilize effort‐based strategies that may lead to self‐control failure, whereas those with better trait self‐control may have developed adaptive techniques that do not rely on the active resistance of temptations, and thus less vulnerable to depletion.^5^ Future research should explore the role of trait self‐control within the depletion effect, and specifically within a sport‐specific context.

Moreover, in light of the findings of the present study, it would be beneficial for researchers to examine potential intervention strategies to reduce the effects of self‐control exertion, and improve subsequent skill‐based performance. Such intervention strategies could include behavioral and cognitive training methods to enhance self‐control;^58^ however, such strategies are yet to be tested in a real‐world sporting performance setting.

## 5 CONCLUSION

The present study provides novel evidence that the prior exertion of self‐control leads to detrimental effects on subsequent sporting skill performance. Furthermore, sustained attention was also detrimentally affected by the prior exertion of self‐control; with this being a potential mechanism to explain the poorer sporting skill performance in the latter stages of the field hockey task in the present study. However, there was no effect of self‐control exertion on cortisol concentrations, suggesting that this is not the mechanism through which prior self‐control exertion negatively affects sporting skill performance and sustained attention. These findings have important implications for those interested in optimizing sports performance, suggesting that coaches and athletes should aim to avoid self‐control exertion before a competitive match.

## 6 PERSPECTIVES

The present study shows that following the prior exertion of self‐control, skill performance (on a field hockey task) is reduced; as evidenced by the hockey players making more errors during the skills task, particularly in the later stages. This increase in errors was accompanied by poorer sustained attention (evidenced by lower accuracy on the RVIP task), suggesting that changes in sustained attention may be the mechanism responsible for the poorer skill performance following self‐control exertion. These findings have very important implications for athletes for whom skill performance is a key element of their overall performance (eg, team sport players), and suggest that these athletes should aim to prevent exerting self‐control prior to competitive matches. Furthermore, this paper demonstrates, for the first time, that prior self‐control exertion negatively affects complex sporting skill performance on an ecologically valid task and thus provides the underpinning rationale for future interventions aimed at enhancing self‐control in athletes for whom optimizing skill performance is important.

## CONFLICT OF INTEREST

The authors declare no conflicts of interest or funding associated with this manuscript.

## Data Availability

The data that support the findings of this study are available from the corresponding author upon reasonable request.
